# Mechanism behind the neuronal ephaptic coupling during synchronizing by specific brain-triggered wave as neuronal motor toolkit

**DOI:** 10.1038/s41598-021-82118-2

**Published:** 2021-02-11

**Authors:** Sajedeh Karami, Mohammad Mahdi Doroodmand, Mahnaz Taherianfar, Amir Mutabi-Alavi, Nahid Nagshgar

**Affiliations:** 1 Department of Chemistry, Shiraz University, Shiraz, Iran; 2 Physiological Division of Department of Basic Science, School of Veterinary Medicine, Shiraz University, Shiraz, Iran; 3 Department of Pathobiology, School of Veterinary Medicine, Shiraz University, Shiraz, Iran

**Keywords:** Biochemistry, Biophysics

## Abstract

Probable mechanism behind the neuronal ephaptic coupling is investigated based on the introduction of “Brain”-triggered potential excitation signal smartly with a specific very low frequency (*VLF*) waves as a neuronal motor toolkit. Detection of this electric motor toolkit is attributed to in-vitro precise analyses of a neural network of snail, along to the disconnected snail’s neuronal network as a control. This is achieved via rapid (real-time) electrical signals acquisition by blind patch-clamp method during micro-electrode implanting in the neurons at the gigaseal conditions by the surgery operations. This process is based on its waveform (potential excitation signal) detection by mathematical curve fitting process. The characterized waveform of this electrical signal is “Saw Tooth” that is smartly stimulated, alternatively, by the brain during triggering the action potential’s (*AP’s*) hyperpolarization zone at a certain time interval at the several µs levels. Triggering the neuron cells results in (1) observing a positive shift (10.0%, depending on the intensity of the triggering wave), and (2) major promotion in the electrical current from sub nano (*n*) to micro (*µ*) amper (nA, µA) levels. Direct tracing the time domain (i.e., electrical signal vs. time) and estimation of the frequency domain (diagram of electrical response vs. the applied electrical frequencies) by the “Discrete Fast Fourier Transform” algorithm approve the presence of bilateral and reversible electrical currents between axon and dendrite. This mechanism therefore opens a novel view about the neuronal motor toolkit mechanism, versus the general knowledge about the unilateral electrical current flow from axon to dendrite operations in as neural network. The reliability of this mechanism is evaluated via (1) sequential modulation and demodulation of the snail’s neuron network by a simulation electrical functions and sequentially evaluation of the neuronal current sensitivity between pA and nA (during the promotion of the signal-to-noise ratio, via averaging of 30 ± 1 (n = 15) and recycling the electrical cycles before any neuronal response); and (2) operation of the process on the differentiated stem cells. The interstice behavior is attributed to the effective role of Ca^2+^ channels (besides Na^+^ and K^+^ ionic pumping), during hyper/hypo calcium processes, evidenced by inductively coupled plasma as the selected analytical method. This phenomenon is also modeled during proposing quadrupole well potential levels in the neuron systems. This mechanism therefore points to the microprocessor behavior of neuron networks. Stimulation of the neuronal system based on this mechanism, not only controls the sensitivity of neuron electrical stimulation, but also would open a light window for more efficient operating the neuronal connectivity during providing interruptions by phenomena such as neurolysis as well as an efficient treatment of neuron-based disorders.

## Introduction

Action potential (*AP*) is the digital electrical signal of neurons that affords essential functions by converting incoming inputs to neuronal outputs alongside their axon^1^. Identification of the nerve cell electrical function according to electrical current flow during AP has been accepted^2^; so characteristic of the AP is important especially for inherent neurodegenerative disorders^3^. It has been found that, ion conductance across the membrane increases dramatically during the AP, while recording from the giant axon of the squid^4^. This discovery was the first evidence that changed in the flux of ions through channels in the membrane that have led to the AP production based on the concentration gradients of Na + and K^+^^5^. The complexity of this effect is further explored when dealing with the neuronal trafficking electrical signals along the neural cord such as the spinal cord, in which the axon is 1 m long^6^. In this event, the trafficking of electrical signals overall the axonal and synaptic terminal is extremely significant^7^.

Understanding the properties of ion channel molecules was greatly enhanced by patch-clamp studies of the electrical potential/current through single channels^8^. Briefly, the patch-clamp technique is a methodology with signal quality and temporal fidelity sufficient to report the synaptic and ion-channel mediated membrane potential changes thereby enabling neurons to compute information affected in brain disorders or by drug treatment^9^. Whole-cell patch-clamp electrophysiology of neurons is also a gold standard technique for high-fidelity analysis of the biophysical mechanisms of neural computation and pathology; however, it often requires plenty of skills^10^.

Following the improvement of this technique, “Blind Patch-Clamp” was raised due to the unclear electrical signal acquisition from the neuron’s membrane. This technique has possessed several advantages: 1—Blind patch clamping simply provides the possibility to the signal recording, directly, in different central nervous system (CNS) regions. 2—This signal recording can be operated at tissue environments and tissue depths with a wide variety. 3—Effect of external activators can be studied in vivo/in vitro using this method 4—Blind patching process is also considered as a reliable method for the intracellular dye labeling studies, 5—The blind patch technique is partially low expensive, especially during comparing with the direct visualizing methods. And 6—The method is simple and flexible during expediting the tissue manipulation. Consequently, the technique seems to be applicable easily for an electrophysiological recording process in different tissues engineering^11^.

According to the previous reports^12^, the plasma membrane is stimulated by the polarity of the actin filament in the presynaptic and postsynaptic regions. This is occurred during the electrical incisive (growing) process by the porosity, fusion pore, fluctuations, channel/synaptic noise, ion charge influx as well as the charge distribution of ions inside and outside the neuron cells^13^. Subsequently, information on the style of the plasma membrane polarization, not only manages the performance of the molecular trafficking toolkits inside the neuron cell, but also may provide an adequate perspective for curing the damaged neuron^14^. To the bests of knowledge, unfortunately, problems such as the lack of adequate compatibility (coherency) between the AP and the modulating electrical stimulus as well as, the electrical activities of the AP have been limited to the only blind and rough estimation of the neural behavior^15^. Accordingly, in this report, for the first time, signal modulation of the hyperpolarization period of the APs as an intrinsic modulating stimulus in the neuron has been investigated through the blind patch-clamp technique.

Controlled stimulation of neurons can be considered as an effective probe for approaching the neuronal disease treatment. This phenomenon has occurred when having enough knowledge about electrical neuronal stimulation^16^. Precise exploring all aspects of the neuronal electricity influential factors can help the neurologists more reliable cure the neuronal diseases. Some of these important factors include modulating waveform (*potential excitation signal, Ψ*), time interval, frequency, and amplitude of the triggering waveform^17^. At this condition, every arrhythmia and deviation from the normal characteristics of this harmony can be considered as the risk factors about the neuronal diseases via the neuronal electrical regulation process^18^.

In this condition, this phenomenon seems to be more important, when dealing with sophisticated neuron electricity such as neural networks. These systems are electrically connected through hypotheses such as direct connecting of neuronal ephaptic coupling^6^. However, precise controlling the neuronal trafficking network with high enough electrical sensitivity has been considered as an important challenge^15^. These problems have led to have very blind control in the neural curing process like neurolysis^17^. To solve this problem, hereby in this report, the mechanism behind the neuronal ephaptic coupling has been introduced during synchronizing by “Novel” and specific “Brain”-triggered very low frequency (*VLF*) waves as a neuronal motor toolkit in detail.

## Experimental

### Reagents and materials

All the chemical reagents were from of their analytical grades. The nutrient media (Stock solution of DMEM/F12.) with l-glutamine, and without sodium pyruvate and sodium bicarbonate (cat. no. 12100-046, Invitrogen) and ham’s F_12_ contains L-glutamine (Cat. no. 21700-075, Invitrogen) in 1.0 L water, were purchased from Sigma-Aldrich Company.

Deionized water (Specific conductivity: 18.2 ± 0.4 MΩ cm, Merck Company) as green solvent for dilution of the nutrient medium to 1/fivefold excess. In addition, B27 supplement (20.0 ± 0.1 ng mL^−1^, 50X, Thermo Fisher Scientific–US). Standard solution of antibiotics penicillin–streptomycin (10.0 ± 0.1%, V/V) was related to the Merck Company. Buffer solution used in the dissection process included 4-(2-hydroxyethyl)-1-piperazineethanesulfonic acid (*HEPES*)-buffer (30.0 mL) protected at cool condition (4.0 ± 0.1 °C) (Cat. no. H-0887, Sigma-Alrdich Company Inc.). The anesthetics drug included Ketamine/xylazine (*KX*) with 80–100 and 10.0–12.5 mg kg^−1^ IP dosage, was from the Sigma-Aldrich Company (Pharmaceutical-grade). The whole stem cell DMEM/F_12_ (Dulbecco's Modified Eagle Medium: Nutrient Mixture F-12) media consisted of 1:1 volumetric ratio of DMEM (high concentrations of glucose, amino acids, and vitamins) and Ham's F_12_ media was purchased from Sigma-Aldrich Company. For cell counting, trypan blue solution (4.0 ± 0.1%. V/V) was from the Thermo Fisher Scientific, U.S. Epidermal growth factor (EGF) (Sigma-Aldrich Company.) to give a final concentration of 20.0 ng mL^−1^ of EGF. In addition, solution of trypsin-ethylenediaminetetraacetic acid (*EDTA*) solution (trypsin–EDTA) with 0.05 ± 0.01% (V/V) concentration and no phenol red was purchased from the Merck Company. Phosphate buffer solution (PBS, pH = 7.0 ± 0.1) with 0.01 molar concentration was form the Sigma-Aldrich Company. Gas solution of CO_2_ (5.0 ± 0.1%, W/W) was purchased from Pars Balloon Company (Shiraz, Iran). Paraformaldehyde (*PFA*) solvent with analytical grade (> 99.9%) was purchased from Sigma-Aldrich Company. Solution of 4.0 ± 0.1% (*V/V*) solution of PFT inside PBS (*PFA-PBS* solution) was prepared for the immunocytochemistry assay.

For the immunoassay analysis, Tween 20 solution was belonged to the Sigma-Aldrich Company. Tween 20 (10.0 ± 0.1%) in PBS (0.01 mol L^−1^) was prepared and then mixed with Trypsin (> 99%, Sigma-Aldrich, 1.0 mg mL^−1^) as serum Inhibitors with volume ratio of 10.0 ± 0.1% (*V/V*) for the NSCs washing process. The primary antibodies included nestin with dilution concentration of 1:500 (1.0 µg mL-^1^), Thermo Fisher Scientific, U.S.) and rabbit polyclonal anti-β-tubulin(III) (1:200, ~ 1.0 µg mL-^1^, ) M01857-1, Boster Biological Technology, Pleasanton CA, USA).

A goat anti-Rabbit IgG (H + L) antibody (conjugated with Fluorescein isothiocyanate, FITC with 1:1000 dilution factor, 1.0 mg mL^−1^), contained with other agents such as PBS (10.0 ± 0.1 mmol L^−1^, at pH: 7.6 ± 0.1), as buffer solution, sodium chloride (NaCl, 150.0 ± 0.1 mmol L^−1^), as electrolyte medium, sodium azide (NaN_3_, 0.05%, W/V), as preservative agent and bovine serum albumin (BSA, 1.0%) as stabilizer was selected as the secondary antibody that was purchased from Arigo Biolabratories Company (ARG54745, Tehran, Iran). This reagent was stored at 2–8 °C inside a refrigerator during production from any prolonged and avoid from exposure to the light as well as any freeze/thaw cycles.

To detect the successive progress in the for detection of differentiated process of the in NSCs by the recommend procedure, the enzyme-linked immunosorbent assay (*ELISA*) kit (also known as human nestin ELISA kit, Catalog # MBS733436) included β-tubulin(III), as a monoclonal antibody and Rat nestin (50.0 ng mL^−1^), that was from MyBioSource Inc. (British Columbia). This specific human assay kit processed characteristics such as competitive assay type, human specificity, 50425 Dalton structural protein, 450 amino acid, as well as figures of merit including detection range of 0.5–10.0 ng mL^−1^, 0.1 ng mL^−1^ sensitivity and 28 °C thermal stability.

### Sterilization process

The sterilization of each equipment was achieved via washing with detergent and water for at least three times. The metal tools were sterilized by pyrolysis during direct introduction to CH_3_OH-air (Industrial methanol, 90%, V/V, Petrochemical Company, Shiraz, Iran) flame using an alcohol burner (15 mL, E-AB300BR, China) for 3.0 min. Further, the sterilization process was achieved by autoclaving at 110.0 ± 0.2 °C (1.80 ± 0.01 atm, humidity: 80%) for 4.0 h using a horizontal high pressure autoclave (Double wall stainless steel, 30.0 L, MCS-XD35J, 35L, MCS-XD50J, China).

### Experimental model and subject details

#### Animals

Two distinct living tissues were used in present study: (1) Differentiated neural stem cells (NSCs) were isolated from the hippocampus of 14-day old embryonic rat (Mouse Embryonic Stem Cells: Protocols & Procedures, Sigma-Aldrich and Merck Companies), and (2) Snail spinal cord (Calendar: Sep. 2018) under controlled conditions in a sterilized lab based on standard guideline (Ch. S. Cohan, J. L. Karnes, and F.-Qu. Zhou, Culturing Neurons from the Snail Helisoma, Method. Cell Biol., 2003, 71, 157–170 and Atlas: 9780123914484: Medicine & Health).

#### Straightforward setup before dissection

Before any dissection, each medium was further sterilized using a reference guideline (T. R. Rosenstock, and R. Iwata, Isolation and Maintenance of Striatal Neurons, bio-protocol, 2018, vol. 8), via heating in a water bath (DK-420, China) at 37.0 ± 0.1 °C for 30.0 min. To accumulate the embryos, the slices were placed in a sterile 50.0 mL conical tubes (3SG700-5, China) with DMEM/F_12_ media. Also, five Petri Dishes with Vented Lids (100 mm × 15 mm, Labnique, China) were needed for stocking the tissues during dissection under similar conditions; the dissection microscope was finally located inside a laminar flow and is sterilized with 70% (V/V) alcohol (Analytical grade, Merck Company).

#### Neural stem cells isolation

Neural stem cells were isolated from the hippocampus of 14-day old embryonic rat (20 ± 1 g) as stated by one's official permitted animal protocol (Neural Cell Culturing Guide, Biotechne, Thermo Fisher Scientific—US). For this purpose, briefly, the rat was positioned from the ground up for anesthetizing via direct injection of the anesthetics drug (0.5 mL) using an insulin syringe (1.0 mL, BD 324704, Iran). After 30.0 min, the rat was sacrificed contained in each the animal moral charter. The embryo cell was separated overall the surgery process according to a reference protocol “Transgenic Animal Technology: A Laboratory Handbook, Books-A-Million, IndieBound, C.A. Ingraharm and C.A. Pinkert, 2nd Ed., Edited by C.A. Pinkert, 2002, Rochester, NewYork), Finally, the cells were cultured in complete NSC medium, supplemented with 5.0 mL DMEM F_12_, meida (1.0%B27, 20.0 ng mL^−1^ epidermal growth factor (EGF ) and 10.0% pen/strep) and used for T_25_, flasks.

#### Neuronal cell counting

Photographic method^19^ was utilized to achieve a cell count, 15.0 mL of the cell suspension was mixed with 85.0 mL trypan blue solution. Lastly, the cells containing at least 25.0 µm thickness were plated to prohibit the glia and endothelial cells. After that using hemocytometer, the cells were calculated to be 2.0 × 10^5^ cells per mL in the stem cell DMEM/F_12_ media.

#### Passaging

The NSCs were passaged using a reported protocole (Neural Cell Passaging Guide, Biotechne, Thermo Fisher Scientific—US)^11^, each medium with suspended stem cells was separated from the flasks, and prepared for subculture with mean cell diamater of 160–200 µm. They were then located in a sterile 15.0-mL conical culture tube (20.0 mL, Thermo Fisher Scientific—US), and centrifuged at 8000 ± 5 rpm using a high speed centrifuge (BM20-1615, Hermele, AH Hermel Candy & Tobacco Co., Nicollet) for 5.0 min at room temperature. After that, the spheres were re-suspended in the trypsin–EDTA solution (0.05%, V/V, 1.0 mL) and incubated at 37.0 ± 0.2° C in a humidified incubator with 5.0% CO_2_. (MyTemp 65 digital incubator, 50.0 mL, China for 4–5 min to eliminate the supernatant. Afterward, an equal volume of the FBS was applied to break the trypsin activity; and then re-suspended in complete neural stem cell medium (1.0 mL). Finally, for evaluation of cell viability, a cell suspension volume and an equal volume of trypan blue dye were mixed. The cell counts were performed under microscope (Viability count = 70%).

#### Neuron network: culture of the stem cells inside micro-channels

**Figure 1:**
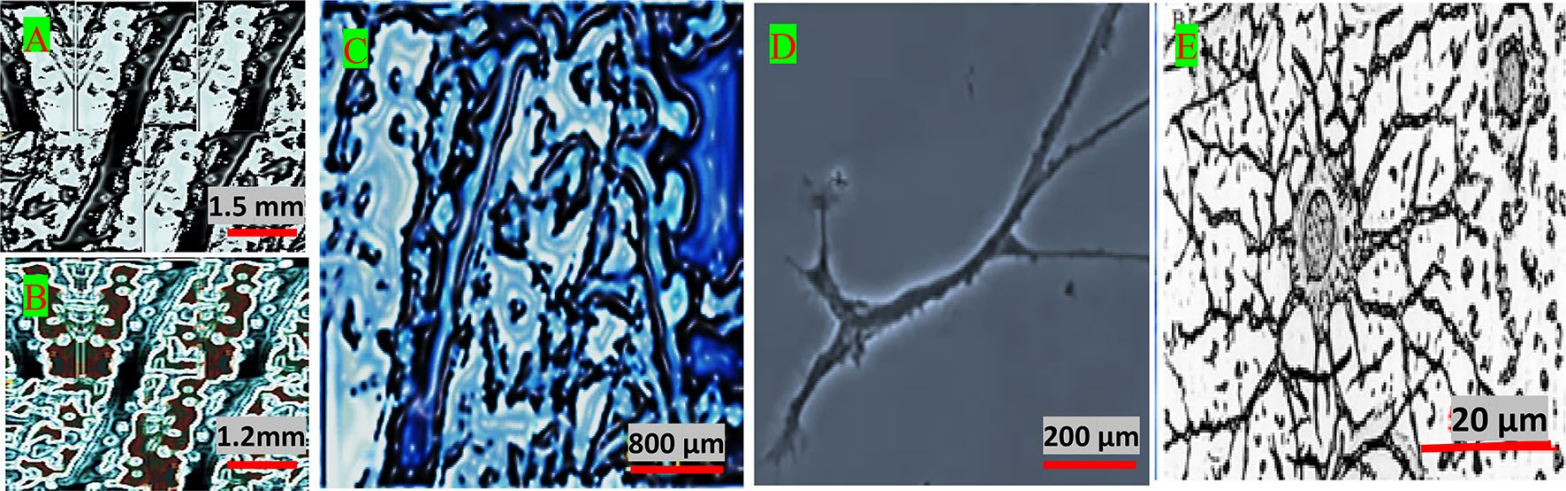
(**A**)–(**D**) Inverted super-resolution fluorescence microscopic images and (**E**) high-resolution cryo-TEM image of the differentiated neuron stem cell immobilized into the micro-channels inside the PTFE Petri Dish.

To evaluate the electrical behavior of the stem cells using a neural cell passaging guide line (Biotechne, Thermo Fisher Scientific—US)^11^, briefly, they were cultured inside a Polytetrafluoroethylene channels (*PTFE*) Petri Dish with lid (55 × 15 mm, LBPD150S, China) and different circular or Y-shaped micro-channels (Average width: 50.0 µm, average depth: 75.0: 50.0 µm, structure: cubic) by laser etching process. Micro-channels with well-defined geometry was achieved via etching (ablating) the internal bottom of the Petri Dish and the laser cutting machine (CO_2_ laser, 1000 W, Rotary Die Board Laser Cutting Machine, Manufacture/Factor & Trading Company, China). The stem cells were formed as the sphere in 4–6 days and after 6 days the neuron rope were differentiated. The fluorescence images with different magnifications were taken by using an inverted super-resolution fluorescence microscopy (2017YFC0110202, China) as shown in Fig. 1A–D. In addition, Cryo-Transmission Electron Microscope (Tecnai G2-30 Cryo-TEM, 30 kV, 30 G2 TWIN, HAAKE Co., Ltd., US) during direct immolation on a Formvar (Agar Scientific Ltd, Stansted CM24 8GF, Verenigd Koninkrij) film (1.0%) in dichloroethane)-coated 3.0 mm copper Cryo-TEM grid (8/2 hole pattern, CDFT823-50, China) was utilized for the direct imaging at the native-like state condition (Fig. 1E).

#### Immunocytochemistry assay

**Figure 2:**
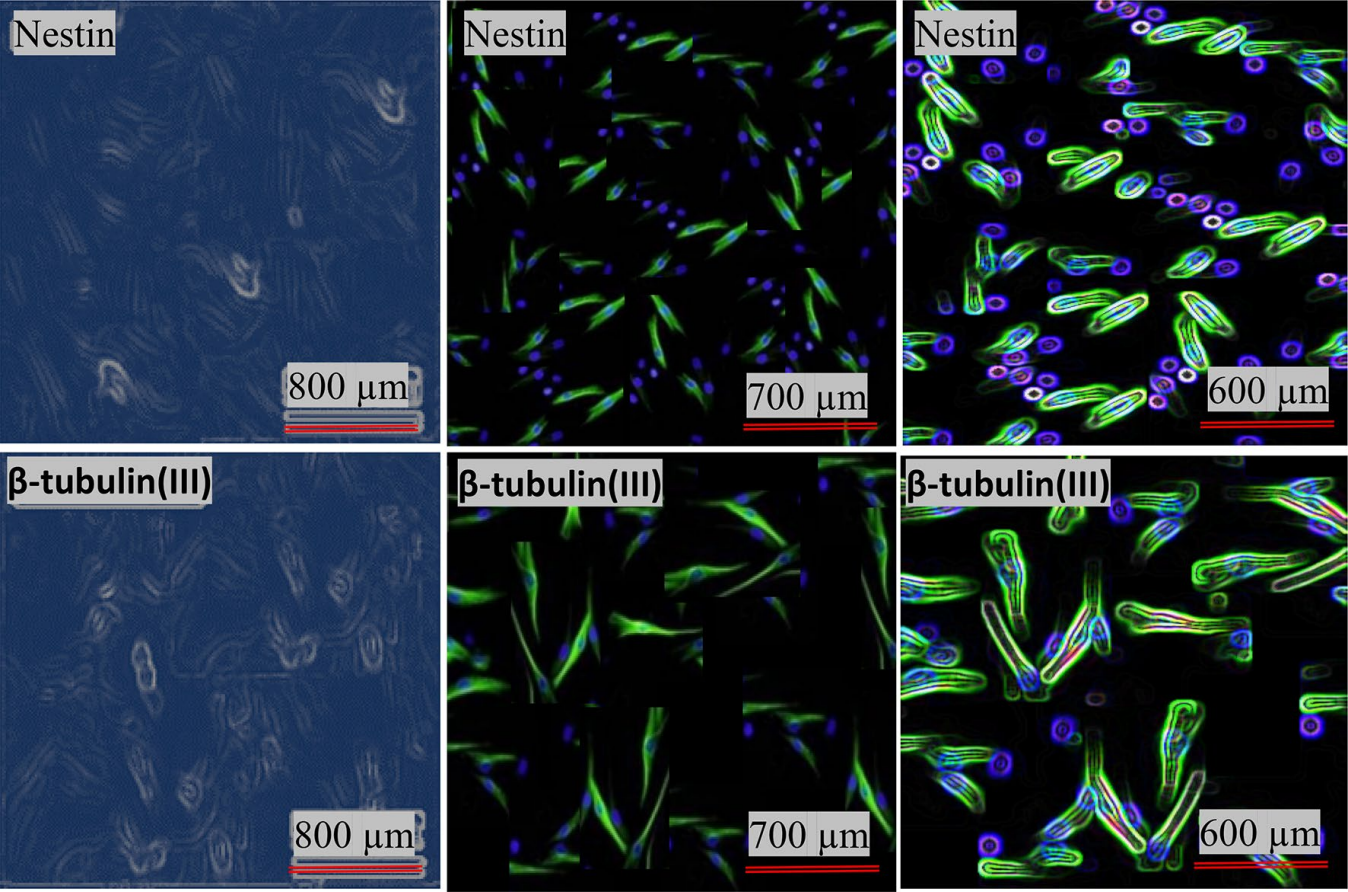
Photographic and fluorescence images of differentiated neuron cells stained with DAPI. Insets: high-resolution images.

To characterize the ability of NSCs differentiation, we used immunohistochemistry test. For this purpose, differentiated cells of 6 days (5.0 mL) were fixed with 4.0% paraformaldehyde in phosphate-buffered saline (PBS) for 15.0 min, then washed three times with PBS. Next, it was washed with Tween-PBS solution in 10.0% serum inhibitor for 60.0 min at room temperature. Cells were incubated overnight at 4.0 °C with primary antibodies including: Nestin (1/500) and rabbit polyclonal anti-bIII tubulin(1/200), formerly washed with PBS and followed by secondary antibody including goat anti Rabbit IgG conjugated with FICT (1/1000), and finally stained with DAPI for detection of cell nuclei (Fig. 2).

#### Nestin and β-tubulin(III) assay analyses

**Figure 3:**
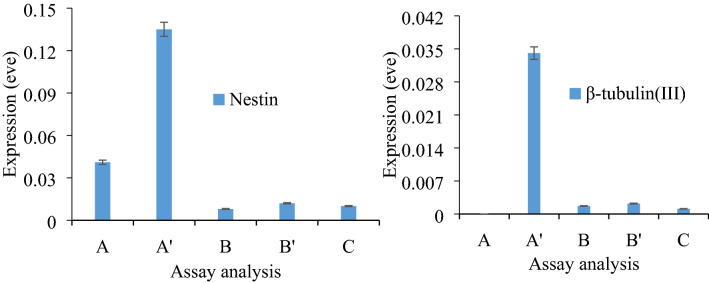
Nestin and β-tubulin(III) assay analyses (50.0 ng mL^−1^) for the differentiated NSCs. The data are the average of 5 replicate analyses, standard deviation: ± 0.08, t-value =  −2.3, *p* value = 0.015 for 95% confidence level and 4 degrees of freedom. (**B**), (**B**′), and (**C**) untreated cells at different time intervals inside the neuron cells (control), (**A**) and (**A**′): treated differentiated cells after 3 days and after passage, respectively and 1:6 ration for the drug extraction and delivery process. Error bars: ± standard deviation (n = 3).

For further identification of different types of NSCs, we used specific antibodies in the nestin and β-tubulin(III) assay; the reliability of the undifferentiated embryonic NCSs during their differentiation to the neuron cells was evidenced by nestin and β-tubulin(III) assay kit based on the reported procedures^20^. The results have been shown in Fig. 3.

To evidence this differentiation, the nestin and β-tubulin(III) assay reagents kit (Pierce, Rockford, IL, USA) with different gene types including A, A′, B, B′ and C were introduced to each neuron medium of NSCs analyses using immunohistochemistry system (Standardizing Immunohistochemistry-NCBI-NIH, room temperature charged couple d device, RT CCD,—cooled color camera, Model#: 3.2.0 W/Attachment, 2.3.0, Diagnostic Instruments Inc., Canon, Japan). This process was therefore used to estimate the color intensity using as ultra-violet (UV–Vis) Spectrophotometry (Varian Cary 4000, Rademarks of Varian, Inc., U.S.) at 495 nm wavelength for each assay probe. This methodology clearly revealed the success of the differentiation process by the recommend procedure reported in protocol^21^.

#### Micro-electrode implant, giga seal condition, blind patch-clamp, action potential detection, curve fitting, and modulation/de-modulation process

Micro-electrode (Epoxy-Insulated Stainless Steel Microelectrode, Catalog #: 572700, A-M Systems Inc., Bedrijf) was selected for the implanting process. The micro-electrode systems were implanted into the neuron membranes by handy or using a three-dimensional (3-D printer, dimension: 30 × 30 × 20 cm, resolution: ± 0.01 mm, LulzBot TAZ 6, USA) and stepper motor (SY42STH47-1206A, Size: 42.3 mm square × 48 mm, China) as a pseudo neurotomy stereolaxic system. This was adopted for culturing in the micro-channels via adjusting the angle and position of the micro-electrode system.

The gigaseal condition was provided based on a reported procedure^22^, briefly, via blind controlling the forward and backward positions of the micro-electrodes linearly using the stepper motor, implanted on the snail’s neuron cell and differentiation NSCs, along with direct measuring the electrical resistance by using an ohmmeter (Digital Multimeter, Fluke 115 True RMS, US). At this ohmic condition, obviously, besides the faraday’s cage, and neuronal condition at 6.42 ± 0.72 GΩ/cm (n = 5) for the cell recognition resistivity, we should filtered most of external random perturbations during the direct potentiometry for sensing the AP using the two micro-electrode systems.

Blind potentiometric patch-clamp method^23^ was selected for AP measurement during implanting a two micro-electrode system with inter-electrode distance of 0.50 ± 0.01 cm at zero current (open circuit) state at the gigaseal condition. All comments of the system were positioned inside a cubic faraday’s cage (20 × 20 × 30 cm, stainless steel, average pore diameter: 0.2 mm) to eliminate high impedance as well as any types of environmental and electrical noise sources. The Ap’s data were then measured using a high-resolution and high-rapid oscilloscope (RTO2000 Series Oscilloscope, up to 6 GHz Bandwidth, R&S-RT, Farnell UK). After that, the data were stored in the random access memory (*RAM*) of the oscilloscope, prior their transferring the data to a PC through the USB port using a written program in Visual Basic (*Version 6, VB*_*6*_) software.

The curve fitting (simulation) of the data was achieved based on “Recursive Curve Fitting” methodology^24^ using “Design Expert” software (A. Dewey, in 21st Design Automation Conference Proceedings, IEEE, 1984, pp. 556–557). To do this, the generated electrical noise was simulated via curve modeling through the functional simulations (“VHSIC Hardware Description Language”, VHDL) to find well-defined waveform (L. I. Maissel, H. Ofek, Hardware design and description languages in IBM. IBM J. Res. Dev. 28, 557–563, 1984).

The AC electrical wave at the VLF region were generated using an external programmable function generator (Brand: DTC, DULARI TRADING COMPANY, DK 1 267 SCH NO 74C VIJAY NAGAR VIJAY NAGAR, Indore—452010, Madhya Pradesh, Two-channel mode, India) using a written VB_6_ program. This functional generator had capability to apply potentiostat electrical voltage at a range between ± 50.00 mV (versus total applied potential).

Blind voltage/current patch-clamp method (like a voltammetry technique^23^) was adopted for applying the electrical AC stimulus using the functional generator with three micro-electrode system as working, counter and pseudo-reference electrodes. Additionally, non-polarization behavior of the reference micro-electrode was controlled at the outer membrane layer of the neuron cells. At this condition, the electrical current also measured by using a current-to-voltage converter electronic circuit and a high resolution—high speed operation amplifier military Seri resistance (Slew rate: < 0.1 µs), OPA2626, Analog Device, China).

The “Modulation” process^25^ was done during triggering (firing) the AP by the curve-fitted (simulated) waveform at a programmable function generator and blind voltage/current patch-clamp method. Whereas, the “De-modulation” process^25^ based on triggering the AP with a 180°-inverted VLF waveform as wholly a complete destructive stimulating potential excitation signal under similar condition.

The frequency domains related to each real and imaginary phases were evaluated via applying “Discrete Fast Fourier Transform” (*DFFT*) algorithm to the time domain (current–time curve) current curve time according to the procedure reported in our previous research^26^.

The “Lissajous Curves” were drawn experimentally via simultaneous generation of each real and imaginary phase by using the programmable function generator and its linear combination by the with an oscilloscope. Besides the waveforms, the situation of this curve was estimated as an appropriate probe for the phase angle recognition process.

#### Snail soma

Snail due to the possessing long neuron system network (length: 5.0 cm)^27^ is considered as one of the most suitable animals for the evaluation of the AP and neuron cell research purposes for testing the AP on the neuron activity of the existent, sever snails (Year: 6 months, sex: male/female) were collected from a jungles area (situation: Golestan, Iran, sampled date: *17th June 2018)*. After anaesthetizing them by Lidocaine (C_14_H_2_.2N_2_O) anaesthetizing reagent (Razi Company, Iran), the nail’s soma was separated by surgery (under sterilized condition based on the method reported in the protocol^28^.

**Figure 4:**
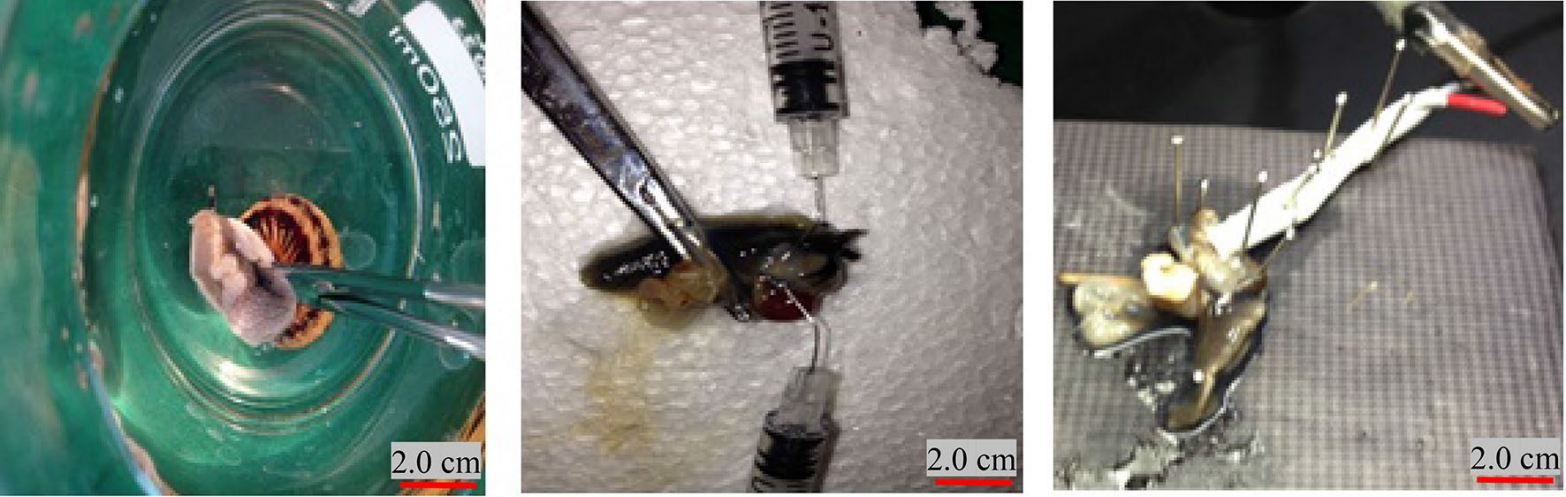
Photographic images of the snail soma at different stages of the in-vitro blind patch-clamp.

The soma was protected in the suitable nutrient medium^27^. Then, the two micro-electrode (OptiMicro Microdissection Needle Electrode, stainless steel, Type: 310), surrounded insulator: Silicone, conductive tip diameter: 1.00 ± 0.01 μm, conductive height: 1.00 ± 0.01 mm, Herdsman Business Park Osborne Park, WA 6017, Australia) system was introduced to the soma with inter micro-electrode distance of 0.25 ± 0.1 mm, and specific resistivity of 6.41 ± 0.13 GΩ cm. The electrical stimulation potentials were applied according to the recommended procedure and the current results were recorded. The photographic images of the snail soma at different stages of the blind patch-clamp are shown in Fig. 4.

#### Spectroscopic analysis

The concentrations of each K^+^, Na^+^, and Ca^2+^ ions during the modulation and demodulation process were measured via direct sampling (1.0 mL) of the nutrient differentiated NSCs medium by using a sampler (LC-4000 Series, China). The samples were then simultaneously analyzed by “Inductively Coupled Plasma” (*ICP*) spectrometer (Varian Vista-MPX CCD Simultaneous ICP-OES brochure) by standard addition method at the selected maximum emission wavelengths.

#### Statistical analysis

Weight estimations of the factors at the two estimated high (+ 1) and low (-1) levels were based on the “Regression” method^29^ using the “Design Expert” software (*Version 11*). The “Simplex” method^30^ was also adopted to evaluate the reliability of the curve-fitted waveforms using this software.

## Results and discussion

### Initial view about the VLF-affecting neuron cells

**Figure 5:**
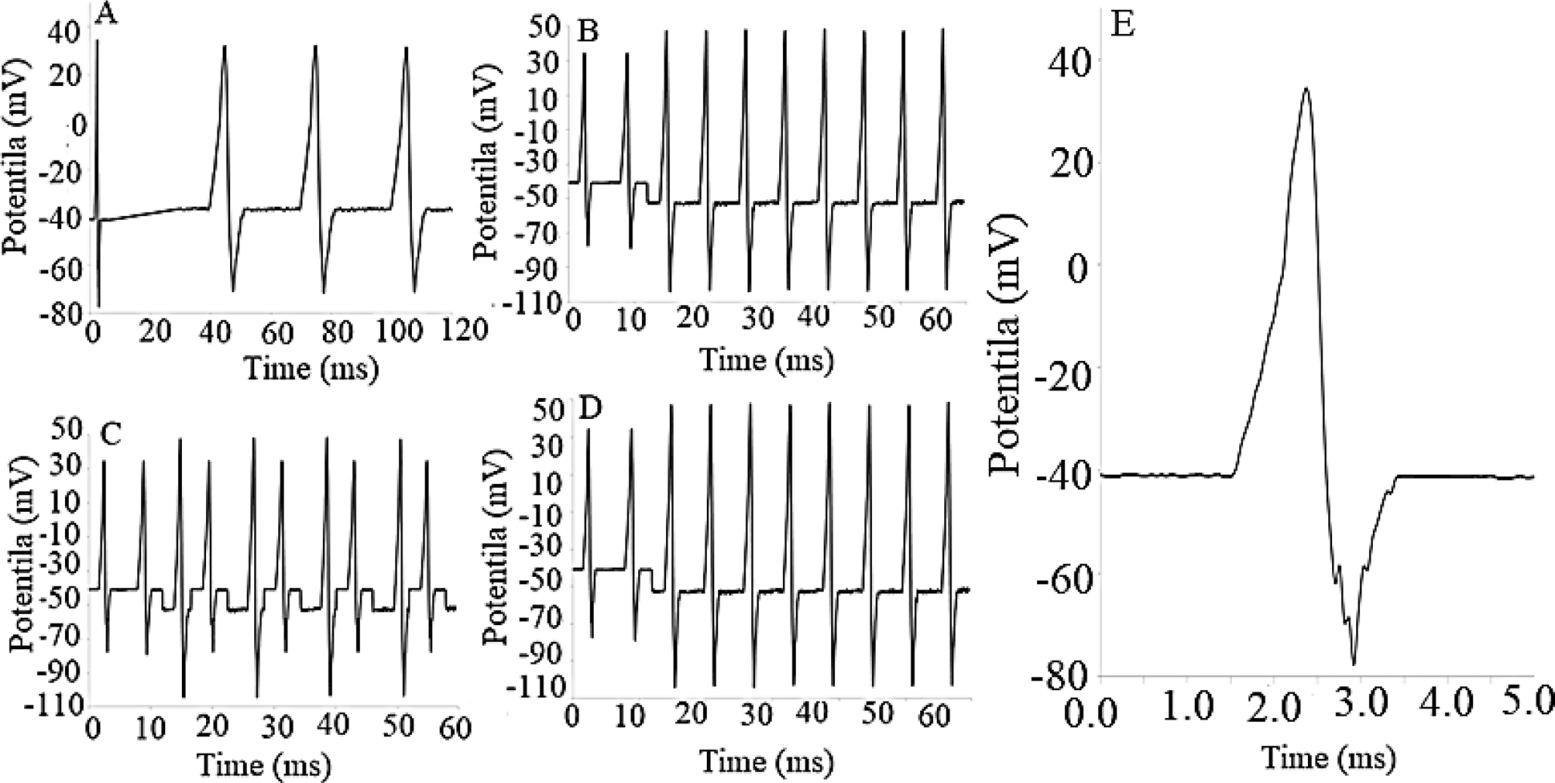
Action potential of neuron snail including (**A**) Control, (**B**) Noise modulated AP, (**C**) Stimulated potential modulation AP, (**D**) reproducible modulation/demodulation during voltage modulation on the AP and (**E**) Specificity of the hyperpolarization zone on the neuron sensitivity. *Note*: The potentials were estimated by potentiometry versus pseudo reference micro-electrode at the giga seal conditions using the two micro-electrode system.

The experimental findings are based on different steps, which are reported in detail as follows:

1. Blind potentiometric patch-clamp method during implanting a two-micro-electrode system onto the neuron membranes. This system was also applied to the two types of snail’s neural cords including brain (1) Disconnected as control and (2) Connected neuron cords under similar conditions. The Aps were then detected and compared in detail using the introduced high-resolution and high-rapid oscilloscope inside the faraday’s cage. The results suggested the intrinsic and selective modulation of the neuron at the hyperpolarization zone (Fig. 5A). The process therefore resulted in appearance of significant shift in the electrical potential of the snail’s brain-connected neuron cords, compared to the control one. This phenomenon pointed to the effective role of the brain for directing this neuronal toolkit behavior probably during formation of a specific potential excitation signal (Waveform) as electrical modulator during evaluation of the snail’s neuron-connected neuron cords.
2. To evaluate for evaluating the effective role of the brain matrix, the electrical potential-time of the neuron cells were traced at a very short time scale (at sub 2 µs). Precise evaluation of the NSCs or snail’s brain-disconnected neuron rope revealed the effective role(s) the external perturbations electrically using the external programmable function generator at the *VLF* region (Fig. 5B). It seems that, brain matrix plays partially similar role for detecting this intrinsic phenomenon. Consequently, a fundamental electrical AP’s modulation process is concluded.
3. Electronically separation of the modulating waveform along with its curve fitting explored a well-defined noisy signal as responsible AP’s hyperpolarization stimulus at the gigaseal condition (Fig. 5B, C), which majorly demonstrated the specificity and its intrinsic auto-generation in the neuron systems (Fig. 5D, E).

As shown in (Fig. 5), presence of electrical resonance between the AP and the noisy signals that generated by the snail’s brain result leads in observing these significant changes in the electrical potential of the neurons’ membrane. This surprising phenomenon also resulted in sensitive and real-time simulation of the NSCs that is considered as the mechanism behind the ephaptic coupling during efficient treatment of neuron-based disorders as according to the smartly microprocessor trafficking toolkit, which roughly introduced before^31^.

### Significance of the hyperpolarization zone perturbation

To evaluate the significance of the detected perturbation at the AP’s hyperpolarization zone, the responses of different AP zones of the AP were detected via averaging the single-to-noise ratio (*SNR*) of different zones during 30 ± 1 (n = 15) sequential (recycled) times. All the results exhibited the specificity as well as the significance and alternate repetition of the VLF noise (Alternative current, *AC*) perturbation only at the “Hyperpolarization” zone of the AP. However, at the initial surface point of view, this process was considered as only a “Simple Noise Phenomenon”, which was seemed to be obvious to be occurred during electrical balancing the neuronal electrical potential by K^+^ and Na^+^ ionic pumping at this hyperpolarization zone. Nevertheless, combination of these small noise perturbations led to see significant change in the potential of the AP (Fig. 5). Consequently, it was decided to precisely characterize the AP’s stimulated AC firing signal in detail using reliable methods.

### Characterization of the AP’s hyperpolarization zone modulation

To estimate the selective and optimum waveform (potential excitation signal), the responsible waveform was detected via recursive “Curve Fitting” methodology using the recommended procedure. The initial rough estimation of the electrical stimulus potential using the programmable function generator after at least (30)^1/2^ enhancement in the signal-to-noise ratio (promotion of the sensitivity) has been reported in Table 1. As a result, curve fitting of these electrical signals resulted in dealing with VLF signal with the electrical characteristics confirming that confirmed the optimized predictions.

**Table 1:** Curve fitting (modeling) of the electrical VLF signals related to the AP perturbations.

V_p-p_ (mV, vs. total applied potential)	Frequency (Hz)	Peak height (mV, vs. total applied potential)	Pulse time (µs)	Relaxation time (µs)	Slope (mV µs^−1^)	Clamped potential (mV, vs. total applied potential)
82 ± 11	157 ± 92	5.7 ± 3.57	48 ± 17	34 ± 7	3.5 ± 1.2	25.0 ± 7

The data are the average of 15 independent analyses ± : Standard deviation.

**Figure 6:**
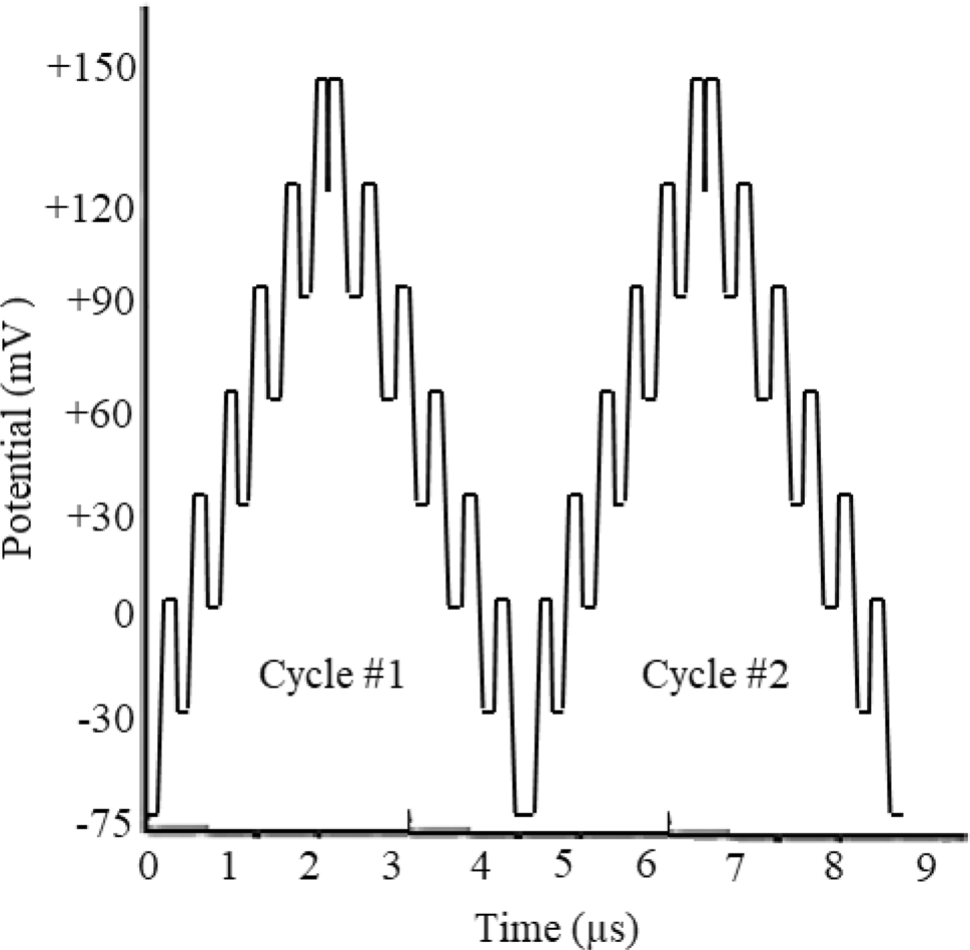
Smoothed curve-fitted waveforms as optimum AP’s potential AC firing stimulus at VLF region at two sequential AP cycles at the hyperpolarization zones. The potentials were estimated by potentiometry versus pseudo reference micro-electrode at the giga seal conditions using the two micro-electrode system.

However, it should be noted that, the significance of this firing stimulus factors (Table 1) was also confirmed by full factorial design with adjusted correlation coefficient (Adj. R^2^) of 0.992. In addition, small deviations with relative error percentages of <  ± 3.25% (n = 15) were detected when comparing the results evaluated experimentally by the curve fitting process to those estimated by “Simplex” method when using eight vertices (using Design Expert software). At these conditions, the potential-time curve of the potential excitation signal (trace diagram) as saw tooth AP’s firing waveform is shown in Fig. 6.

### Similar correlation between the AP’s hyperpolarization zone mode of the snail spinal cord and the differentiated NSCs during firing the AP with the AC waveform at optimum condition

To further evaluate the electrical source of this stimulus waveform, modulated current and/or voltage dependency of the modulating waveform was characterized using patch-clamp method on the NSCs, immobilized (cultured) inside the micro-channels^11^. based on different geometric patterns (templates such as circle, triangular, @, star, etc., https://cad-block.com/277-pattern-lines.html, accesed on 2nd June 2020) with various dimensions ranged between 0.2 and 50.0 mm length and 50–700 µm thickness as channel’s characteristics by a computer-aided software (AutoCAD, 2019, Microsoft), designed in by using a cutting laser (Type: Yag, QL-YC3015, Quick_laser, China).

**Figure 7:**
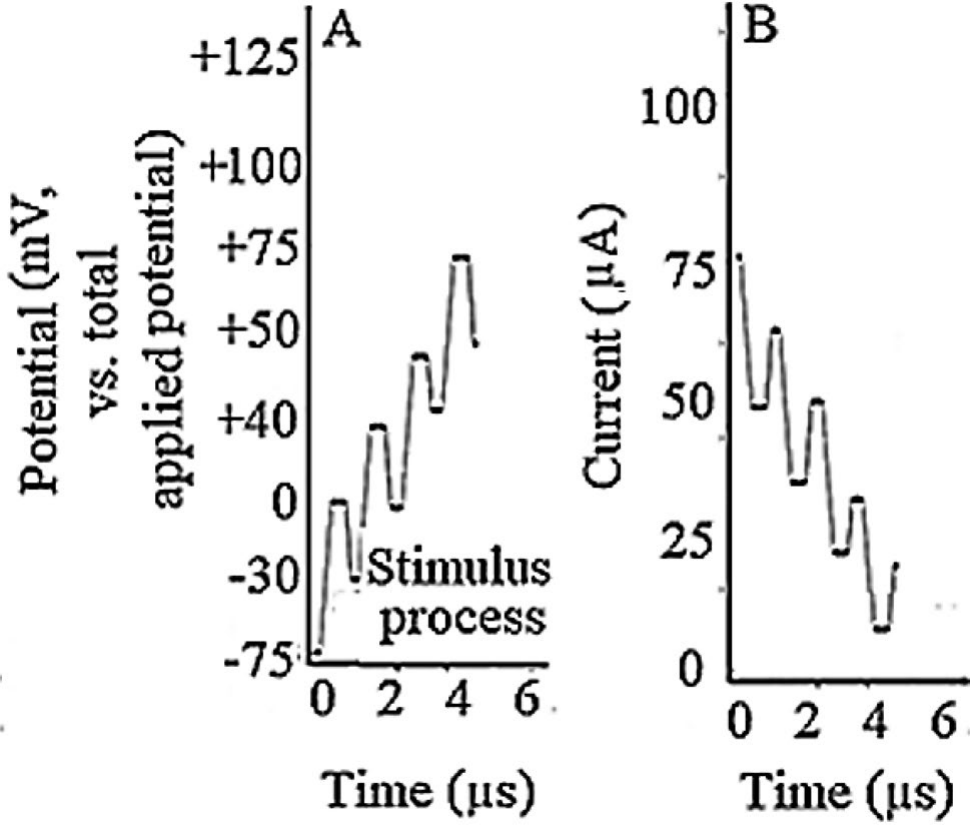
Effects of (**A**) applied optimum waveform to the differentiated NSCs that resulted in (**B**) measuring free induced decay of the electrical current using blind voltage/current patch-clamp method.

To further assess the correlation between the AP’s hyperpolarization zone mode of the snail spinal cord and the differentiated NSCs during firing the AP with the AC waveform at optimum condition, blind voltage/current patch-clamp method was selected using the three implanted micro-electrode system to the differentiated NSCs. The result (Fig. 7A) pointed the main point of this research is the similarity and significance of the experimentally detected AP’s triggering waveform during comparison with that applied to the differentiated NSCs. At this conditions, the current–time response after switching off the triggering (firing) modulation consequently led to have “Free Induced Decay, Fig. 7B” of the electrical currents until reaching to zero current condition, strongly approved the influence of the modulation process even on the high impedance ephaptic coupling of the neuron systems.

### Sequential de-modulation of AP by the introduced AC firing stimulus

**Figure 8:**
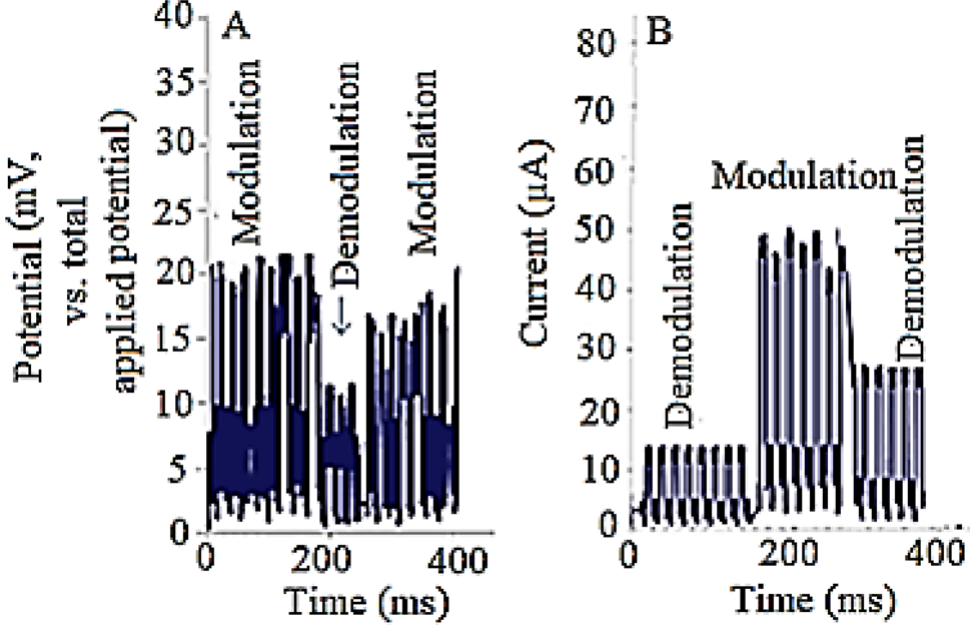
Effects of different sequential AP’s de-modulations on the (**A**) electrical potential and (**B**) measured electrical currents during in-vitro analysis of snail’s brain-connected spinal cord using bliny potentiometry (at giga seal condition) and current-based patch-clamp method.

To additionally further analyze the effect(s) of this AC firing (triggering) signal on the AP, effect of different sequential de-modulations of the AP of a brain-connected spinal cord was evaluated in detail by the blind voltage/current patch-clamp technique (Fig. 8).

As shown in (Fig. 8), based on the introduction of the curve-fitted waveform that intrinsically is triggered and controlled by the “Brain” system of the alive animal. This result was based on the controllable appearance and disappearance (turning On/Off) of the sensitive electrical current (at µA level) during (1) following the electrical signals of the snail spinal cord at three cases: (1) Body-connected and disconnected (separated) modes under similar conditions, (2) Appearance of electrical current when stimulating the isolated snail’s spinal cord by the introduced VLF waveform and (3) Disappearance of the electrical current during stimulation stimulating of a snail’s body-connected spinal cord or differentiated stem cells by the − 180° inverted VLF waveform as the wholly destructive stimulating potential excitation signal. These results, not only pointed to the reliability of the introduced stimulating waveform but also revealed the importance of the brain system as well as the intrinsically occurrence of this process inside the neuronal system networks.

### Bi-directional ephaptic coupling during synchronizing by the AP firing waveform

**Figure 9:**
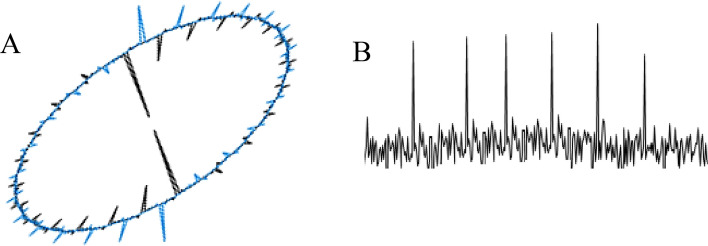
Lissajous curves showing the real and imaginary frequency domain during applying DFFT algorithm on the current–time domain of snail’s spinal cord (**A**) before and (**B**) after applying the de-modulation potential by blind voltage/current patch-clamp.

To answer this question “Why this sensitivity electrical current does not polarize the neuron cells along a spinal cord?”, the electrical correlations between axon and dendrite were evaluated in detail. For this purpose, The *DFFT* algorithm was applied to the electrical potential curve as a time-domain signal (Fig. 7A). In this condition, applying this algorithm, resulted in simultaneous appurtenance of two real (positive amplitude) and imaginary (negative amplitude) with 270° ± 5° (n = 10) phase angle. The Lissajous curve was shown in Fig. 9A; whereas, only one real phase was detected in the absence of any AC firing at the AP hyperpolarization zone (Fig. 9B).

The risibility of this process was further evaluated when (1) detecting the same behavior for the brain—connected,—disconnected snail neuron cords in a snail, (2) successive observing this phenomenon during electrical firing the differentiated NSCs, and (3) controllable collapse of the current sensitivity during AP’s de-modulation with each real or imaginary waveforms. Consequently, regardless of using the “Imaginary” phrase for the reverse electrical current, this waveform has existed in the neuron cords. Besides the brain, the “Microprocessor” behavior of the neuron cord is therefore concluded during precise focusing on this intrinsic phenomenon.

### Microprocessor behavior of the neuron cord

As clearly exhibited (Fig. 9), despite the only unilateral direction of the current vector, two bi-directional electrical current vectors with the same positive and negative amplitudes for each forward and reverse electrical current vectors, respectively, were detected. At this condition, electrical rope with small “Electrical Resistivity” was concluded. As the result, this effect emphasized the higher importance of low resistance ban-gap electrical connections, compared to the high resistance ephaptic coupling. Besides, observation of 270° ± 5° (n = 10) for the phase-amplitude revealed the harmony of these two current vectors during sequentially changes in the initial phase angle versus time. Therefore, continuous neutrality (zero overall net charges) was detected during electrical current flow along the neuron ropes. These shreds of evidence, therefore, revealed the “Motor Toolkit” Behavior of the neuron system during focusing on this electrical firing phenomenon.

### Motor toolkit behavior of the neuron system

Curve fitting of the electrical currents vectors at different simulations positions on the signal’ neuron cord by the blind patch-clamp method, pointed to the probably effective role of the initial phase during controlling of the traffic in the neuronal system. Subsequently, the motor toolkit behavior of the neuron system is majorly concluded during the electrical traffic controlling process. This process is attributed to a hypothesis called “Pseudo Resonance”.

### Pseudo resonance between brain and neuron cords

In this study, pseudo resonance behavior between brain and neuron cords was also evaluated in detail. This hypothesis was based on focusing on the exponential approach of the electrical current–time curve to the highest sensitivity (current intensity) as steady-state condition during electrical current signal averaging up to 30 ± 1 (n = 15) sequential AP’s firing cycles (Fig. 6), depending on the simulation of the neuron systems. At this condition, significant sensitivity up to at least 1000 (n = 5) was observed during the AP’s modulation by the VLF waveform as a neuronal firing signal. This hypothesis was ascribed to the Ca^2+^ pumping as a responsible instructor.

### Instruction behavior of the Ca^2+^ pumping

To investigate the instruction behavior of the Ca^2+^ pumping, the nutrient medium around the differentiated NSCs was sampled analyzed by the ICP spectrometry during following the Na^+^, K^+^, and Ca^2+^ ions by the standard addition method. Based on the results, no significant change was observed for the molar contains Na^+^ and K^+^ ions during at least 10 independent firing ignitions of the NSCs. In addition, no major Ca^2+^ formal concentration gradient was detected under similar conditions. This condition was therefore selected as control; whereas, de-modulation of real-phase of the electrical current resulting in 12.3 ± 0.4 (n = 3) % reduction in the Ca^2+^ concentrations that pointed to the hypocalcemia process. Also, the reverse result (i.e. hypercalcemia) as large as 10.6 ± 0.5% (n = 3) was measured during the de-modulation of the imaginary phases. These results, therefore, exhibited the importance and responsibility of the Ca^2+^ pumping at the AP’s hyperpolarization zone during the sequential hyper/hypocalcemia process that behaved as previously introduced as a Ca^2+^ sensor^6^.

### Proposed mechanism and modeling

Details of the proposed mechanistic behavior of the electrical current in the neuron system are reported in Table 2.

**Table 2:** Summary of the adopted experiments for approaching to the mechanistic behavior of the neuronal electrical signals.

No	Method	Comments
1.	Neural rope differentiated stem cell during immobilization inside microchannels (in-vitro analysis)	The same results were observed during in-vitro ignition of the neural rope on the lab scale using the high-resolution oscilloscope
2.	Snail’s spinal cord voltage-based patch-clamp (in-vitro analysis)	Partially the same correlation was detected after physically disconnecting the connection between the different rates brain and the spinal cord through the first bead of the snail’s neck
3.	Correlation between the short-time signal and Ca^2+^	To investigate the mechanism behind the short-time signal, the nutrient medium around the stem cell that was differentiated by the proposed mechanism was analyzed by the ICP spectrometry during following the Na^+^, K^+^, and Ca^2+^ ions, that pointed to the sequential hyper/hypocalcemia process

**Figure 10:**
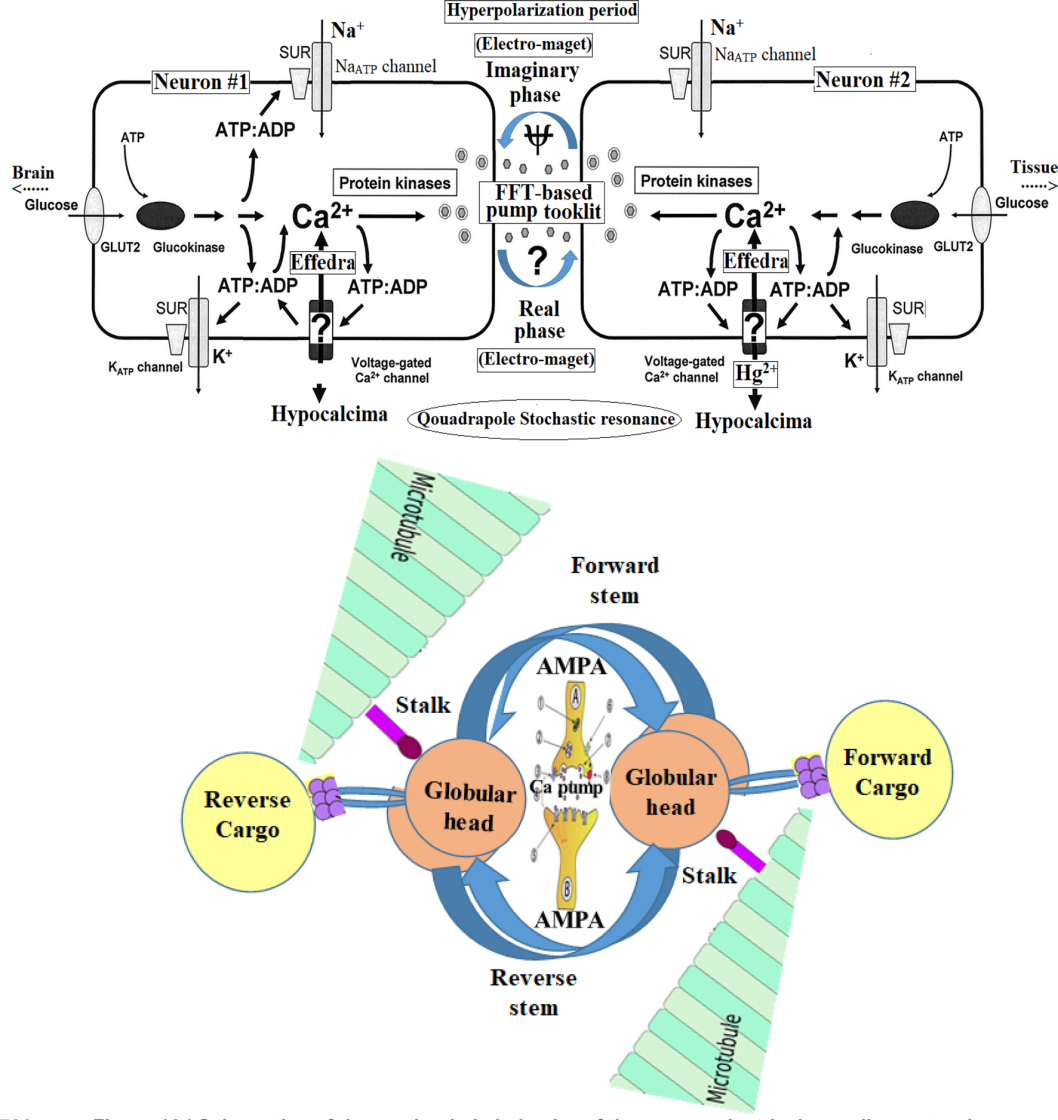
Schematics of the mechanistic behavior of the neuron electrical recycling network.

Based on these pieces of evidences, the schematic view of this mechanistic behavior of the neuron electrical recycling network has been shown in Fig. 10.

**Figure 11:**
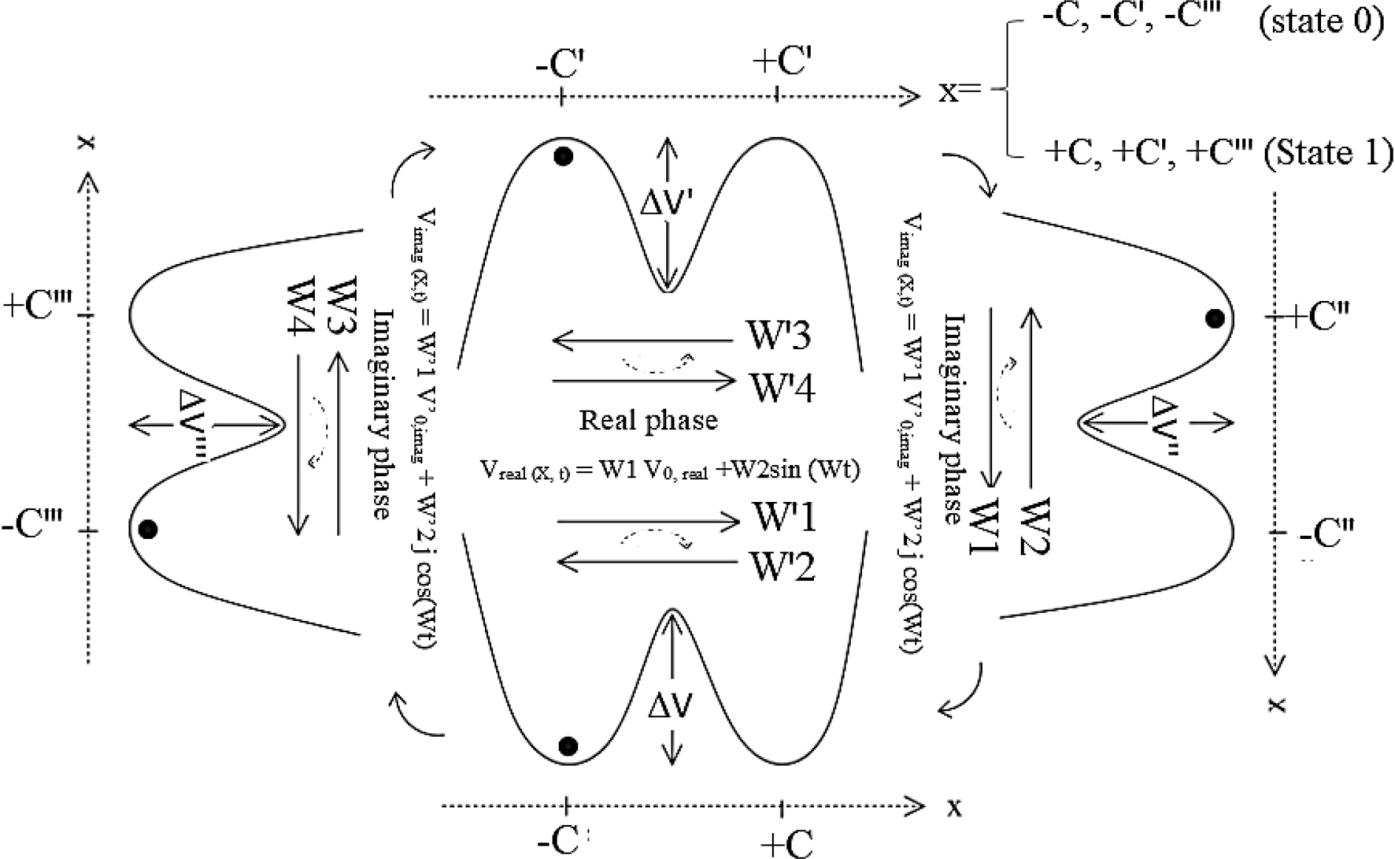
Modeling of quadrupole well potential V_0_(x) inside the neuron recycling network. W_1–4_ revealed to the weight of each current vectors.

Based on this suggested mechanism (Fig. 10), it also seemed that small electrical conductance of the neuron, as well as a low signal-to-noise ratio, causes an intrinsic problem during the inter-neuron communication. According to the literature, a growing number of studies indicated that the noise generated by randomly channel opening limits neuronal response reliability^32^, influences the energy efficiency of channels^33^, and constrained the physical dimensions of axon diameters^34^. Conversely, noise can have a beneficial role by enhancing the detection of weak periodic signals via pseudo-stochastic resonance mechanism^35^ or via enhancing subthreshold oscillations^36^. Consequently, it seemed that the magnitude of the channel noise is under evolutionary pressure in the biological system seeking an energy-efficiency coding system^37^. The channel noise is originated from the neuron channel I_h_^38^ bearing in mind that, (1) channel noise is greater compared to the voltage-dependent channel of the I_h_, (2) channel noise increase linearly with the channel number, and (3) channel noise would be expected to lead to larger voltage noise in a small compartment in which I_h_ channel densities are maximal^39^. Consequently, the channel noise can stimulate electrical channel activity (i.e. I_h_)^40^. This can be adopted as an appropriate key factor for operating the electrical behavior in the neuron cells. The detail of the recommended model has been shown in Fig. 11.

Based on the recommendation, model a quadrupole with 270° ± 5° (n = 10) phase gradients has been investigated for simulations bi-directional electrical communication of the neuron signals.

### Probable future perspective of this phenomenon

However, investigation of partially more improved reproducibility for the electrical currents during at least 50 sequential data acquisition on differentiated NSCs with a relative standard deviation of 4.51 ± 0.34% during using the AP’ firing stimulus process. Whereas, this value was expected to be 15.27 ± 0.96% (n = 50) in the absence of this AP triggering phenomenon. As exhibited, stimulation of the neuron system based on this mechanism not only controlled the electrical sensitivity of neuron electrical stimulation but also would open a light window for more efficient operating the neuronal connectivity during providing interruption by phenomena such as neurolysis as well as an efficient treatment of neuron-based disorders. However, in-vivo analyses are needed for having major confidence about these probable future perspectives of this phenomenon.

### Ethical considerations: compliance with ethical guidelines

The animal handling was conducted according to the Ethics Committee for” Animal Experiments at Shiraz University” (71454).

### Ethics committee

This studied was admitted and approved by the ethics committee of the Shiraz University Consul.

### Arrive guidelines

The study was carried out in compliance with the ARRIVE guidelines.

## Conclusion

The reported result reveals that the alphabet of the neuron as a remarkable bio-processor was detected via a novel patch-clamp methodology simply by implanting micro-electrodes to the stem cells or chemically using an ultra-trace injection of the regulating reagents to the neuronal system. In addition, for further safety of the electrical regulation process, optimizing the magnetic pulses results in having a regular current transition process. To the bests of our knowledge, this article is acknowledged as the first report in which a novel and applicable mechanism are reported for the AP performance of the neural system. However, so far plenty of in-vivo experiments have been approved about this process, but after finishing the in-vivo case studies, the in-vivo results will be reported according to our future reports. All these results recommend the reliability and practicality of this method for better dealing with neural diseases.
